# To Dr. Bradley

**Published:** 1801-11

**Authors:** Jonathan Stokes

**Affiliations:** Chesterfield


### TO DR. BRADLEY.

DEAR SIR,

IT gives me great pleaſure to find from your Note at p. 219, that "ingenious artiſts are now at work, in the hope of being able to give accurate repreſentations of the true and ſpurious" Cow-pox, or, as I have propoſed to name them, the *Vacciola ſcutellata* and *leproſa*, as nothing within my ſphere of obſervation has tended to retard the progreſs of the new inoculation ſo much as the difficulty which inexperienced inoculators find in learning to diſtinguiſh the two diſeaſes which unhappily the vacciolous virus is capable of producing. Theſe artiſts will, I hope, colour their plates by hand, as the botanical artiſts do; for if they do not, I am fearful they will not afford much inſtruction to the young Cow-pox inoculator. The medical and the unprofeſſional practitioner ſeem here on a level, for out of eight inoculators who omitted to re-inoculate patients who had had the Vacciola leproſa, four were regular ſurgeons, and a fifth a medical practitioner who had been in the habit of inoculating with Variolous virus. Of their eleven patients, nine took the Small-pox, two died of the Small-pox, and from one of them the Vacciola leproſa was communicated by inoculation to another patient. This laſt was re-inoculated, and was infected with Vacciola ſcutellata; but the patient from whom the virus of the Vacciola leprofa was taken, owing to the prejudices of its parents, now lives expofed to the danger of being carried off by the natural Small-pox.

I remain, &c.

*Chesterfield,* 3 *Oct.* 1801. JONATHAN STOKES.

